# Disability, spirituality and the politics of belonging in postcolonial Zimbabwe

**DOI:** 10.4102/ajod.v15i0.1720

**Published:** 2026-01-29

**Authors:** Nomatter Sande

**Affiliations:** 1 Research Institute for Theology and Religion, College of Human Sciences, University of South Africa, Pretoria, South Africa

**Keywords:** disability, Zimbabwe, spirituality, citizenship, Ubuntu, inclusion, Marco Antonsich

## Abstract

**Background:**

In Zimbabwe, disability is defined and explained from a cultural, African indigenous religion and spirituality. These perspectives are sources of exclusion of persons with disabilities (PWD) from important social positions, economic empowerment, rites of passage and ceremonies.

**Objectives:**

This study explores the politics of disability and belonging in Zimbabwe.

**Method:**

Using Marco Antonsich’s conceptualisation of place-belongingness and the politics of belonging, data are collected through qualitative desktop methodology, which explores how people with disabilities in Zimbabwe make emotional connections with communal spaces while being excluded from them.

**Results:**

The study findings show the struggles for PWD to belong to religious or spiritual practices and cultural and economic spaces in Zimbabwe. The conventional concept of Ubuntu, which calls for interconnection of humans, provides hope to include PWD as part of the community. Disability in Zimbabwe is not one thing; the experiences of PWD are influenced by overlapping identities (such as gender, class and religion). The legacy of colonialism is visible in the spatial exclusion of the PWD, especially in the urban informal settlements, which sustain the pre-existing trends of exclusion.

**Conclusion:**

The study concludes that belonging and belongingness of PWD should be within the grassroots movements and Ubuntu ethics, which encourage humaneness.

**Contribution:**

The study contributes to policy changes informed by decolonised disability justice, participatory governance and the integration of Ubuntu principles to create a more inclusive citizenship and a stronger communal belonging for people with disabilities in Zimbabwe.

## Introduction

Disability in postcolonial countries such as Zimbabwe should be understood from multiple factors like colonial histories, spiritual cosmologies and neoliberal governance rather than only as a medical or social term (Grech 2015:45; Meekosha 2011:3). This article explores how Zimbabwean persons with disabilities (PWD) negotiate inclusion among these conflicting narratives using Marco Antonsich’s (2010:645) dual paradigm of place-belonging (emotional links to community) and politics of belonging (institutional gatekeeping).

This study uses belonging (place-belongingness and politics of belonging [this will be discussed in more detail]) as a theoretical framework to show how PWD negotiate spiritual reverence, colonial era marginalisation and neoliberal neglect (Antonsich 2010). Disability resists one-sided classification. Crenshaw (1989) and Dube (2020) point out that the impact of intersecting elements like gender, education and socioeconomic level moderates its lived reality and provides uncertainty that questions the strict binary of exclusion and inclusion. The Shona and Ndebele people’s indigenous cosmologies view disability as caused by ancestors. The ancestors reserve the right for integration and exclusion depending on how the living ones are behaving. Although PWD could be revered as spiritual intermediaries, this position is nonetheless conditional depending on their use in preserving cosmic equilibrium (Chataika 2012; Dube 2020). Persons with disabilities are often scapegoated as spiritual contaminants and exploited for labour. According to Mpofu (2016) and Mbiti (2024), spirituality weaponises belonging, and this reduces the disability to a negotiable identity rather than an inherent right (Mbiti 2024; Mpofu 2016).

The African ethic of community interdependence (*munhu munhu nekuda kwevanhu*: a person is a person through others) and Ubuntu (*unhu*) have the potential to encourage PWD belongingness. Recent scholarly work has expanded Ubuntu’s role as a basis for inclusive social policy in postcolonial contexts (Mugumbate 2020). Besides the cultural, religious, and spiritual explanations of disability, the medical perspective is also prominent in Zimbabwe. The medical model, which defines disability as an individual deficit, entrenches exclusion. After independence, the medical model persisted: the *1992 Disability Act* gave medical rehabilitation top priority above social integration, hence sustaining what El-Lahib (2015:615) describes as ‘charity-model paternalism’.

Although the postcolonial constitutional protections, notably Article 22 of Zimbabwe’s 2013 Constitution, guarantee disability rights, structural inequalities persist. With rural areas disproportionately impacted by service inadequacies, United Nations Children’s Fund (UNICEF) (2022:15) states that just 12% of Zimbabwean people with disabilities received school or formal employment by 2017. This conflict between progressive laws and lived reality emphasises citizenship as a disputed territory in which legal acknowledgement coexists with daily erasure. Since the 1990s, neoliberal austerity policies have further privatised welfare, thereby characterising disability inclusion as a financial burden rather than a social responsibility. This neoliberal logic hides the need for structural fairness by commodifying care and romanticising Ubuntu as the remedy.

Intersectional marginalisation exacerbates these difficulties. Disability exclusion interacts with many different identities; it is not one thing (Dube 2022). Using Kimberlé Crenshaw’s (1989:140) idea of intersectionality, this section looks at how class, gender and sexuality compound marginalisation for Zimbabwean people with disabilities. For instance, LGBTQ+ people and women with disabilities are prone to exclusion because of patriarchy and heteronormative standards within African contexts. Also, Section 73 of Zimbabwe’s Criminal Code criminalises same-sex intercourse, thus depriving LGBTQ+ people with disabilities of legal recourse and human immunodeficiency virus (HIV) preventative treatment (Muchegwa 2023). Citizenship is stratified as a result of these injustices, which establish spiritual and legal belonging based on obedience to heteronormative rules that govern able-bodied individuals.

Although stressing inter-ministerial collaboration, Zimbabwe’s 2021 National Disability Policy reflects colonial-era paternalism. It excludes Organisations of Persons with Disabilities from decision-making, hence reproducing top-down government (Ndlovu 2022). Thus, transformative inclusion should challenge bureaucratic inertia inherited from colonial administrations by elevating traditional practices, including *dandaro* [community conversations] in policymaking (Ndlovu 2021). For example, changing Article 22 to require accessibility audits of public infrastructure might help to solve physical exclusion; yet such policies are still unrealised.

A brief overview of the literature, particularly those which focus on disability, spirituality, and belonging in postcolonial contexts, shows the role of social and political factors in aspects of belonging. Advocating instead for social and political interpretations of exclusion, disability studies researchers criticise the shortcomings of the medical model (Oliver 1990:22; Shakespeare 2013:15). Postcolonial academics such as Chataika (2012:387) contend that institutionalised segregation is a legacy carried on in post-independence laws.

### Belonging as a theoretical concept

Belonging, as Marco Antonsich (2010:645) proposes, consists of two dimensions:

- Emotional connection to a community grounded in common identity, rituals or spaces; for example, Shona kinship networks are known as place-belonging.
- Politics of belonging: institutional systems (like laws and regulations) controlling inclusion and exclusion, usually favouring able-bodied, heteronormative standards.

While politics of belonging alludes to institutional exclusion (e.g. colonial practices), place-belongingness describes emotional attachments to community (e.g. Shona kinship networks) following Antonsich (2010). In Zimbabwe, disability disturbs both: laws denying citizenship rights and spiritual functions that may give belonging. Disability affects both dimensions: while colonial-era rules impose exclusionary politics of belonging (e.g. *1959 Mental Diseases Act*), spiritual narratives may give place-belongingness (e.g. *mhondoro* roles).

#### Marco Antonsich’s belonging versus exclusion

Marco Antonsich’s idea of belonging, which combines place-belongingness and the politics of belonging, offers a comprehensive framework for investigating the complex experiences of PWD in Zimbabwe. This dual perspective explains how cultural, spiritual and sociopolitical forces shape the contested domains of acceptance and marginalisation that PWD face.

#### Place, belongingness and the politics of belonging

In the Shona and Ndebele traditions, disability frequently overlaps with spiritual narratives, resulting in a complicated tension between reverence and stigma. Children with congenital disabilities may be referred to as *mhondoro* [spirit mediums], bestowing upon them a revered position as intermediaries between the ancestral and human spheres (Chataika 2012). This spiritual agency cultivates a sense of belonging, integrating PWD into the community’s fabric. The notion of Ubuntu, highlighting communal care and interdependence, is seen in kinship structures such as *zvigure* [extended family], when communities consolidate resources to assist PWD (Marongwe 2019). These techniques foster emotional connections to communal areas, establishing PWD as essential to social unity.

This sense of belonging is, however, fleeting. In times of tragedy, such as droughts or epidemics, spiritual tales can capitalise on vulnerability. Persons with disabilities may be unfairly branded as harbingers of death or held responsible for blocking rains, weakening their sense of belonging and community (Dube 2020; Gunda 2020). Failures in urban infrastructure, such as Harare’s inaccessible public transport system, reduce a sense of belonging by physically removing wheelchair users from social places (Ndlovu 2022).

Antonsich’s (2010) articulation of the politics of belonging emphasises how power relations delineate citizenship and govern inclusion or exclusion. This approach is extended here to accommodate the ambiguities of disability, where belonging is neither entirely granted nor rejected but negotiated via overlapping identities and systematic inequalities (Mpofu 2016; Shakespeare 2013). The colonial period established biological frameworks that defined disability from a medical perspective. The colonial legacy continued in post-independence policy, which favoured institutionalisation rather than community integration (*Disability Act, 1992*). Organisations such as the Jairos Jiri Association, albeit offering vocational training, unintentionally sustained isolation, reflecting colonial paternalism (Mpofu 2003).

The systematic eradication of indigenous knowledge systems worsens this isolation. The omission of Shona folktales with PWD characters from educational curricula exemplifies the epistemic violence that denies PWD cultural recognition and representation (Ndlovu 2022). This erasure has an impact on both individual identity development and society’s attitudes towards disability, sustaining discriminatory standards. The curriculum exclusion resonates with earlier critique of colonial education policies which systematically marginalised children with disabilities (Chimedza 2003).

Current legislation and practices reveal the complex politics of belonging for Zimbabwe’s PWD. Despite the constitutional protections outlined in Section 22 of the 2013 Constitution, significant implementation issues persist. Persons with disabilities are badly marginalised in exercising their voting rights because of inadequately funded disability boards, insufficient government facilities and a lack of Braille ballots during elections (Zimbabwe Electoral Commission [ZEC] 2023). These institutional constraints reflect a politics of belonging that associates full citizenship with able-bodied norms, marginalising those who differ from this standard.

#### Layered marginalisation: Gender, class and LGBTQ+ identities

Antonsich’s approach becomes more complex when viewed intersectionally, revealing how different elements of identity and oppression influence sensations of belonging. Women with disabilities and LGBTQ+ people face increased marginalisation as a result of interlocking forms of prejudice. Women with disabilities in Epworth are more vulnerable to sexual abuse, exacerbated by communication barriers that limit their access to social services and judicial institutions (Musasa Project 2022). LGBTQ+ individuals with disabilities confront the compounded challenges of ableism and homophobia, frequently encountering extortion and exploitation linked to societal biases (Gunda 2020). These intersectional experiences highlight the stratification of belonging across various identity dimensions, hindering the journey towards complete social inclusion.

The economic implications of belonging shed light on the difficulties faced by PWD in Zimbabwe. Hyperinflation and economic instability have harmed PWD, reducing social support and increasing reliance on limited government aid systems. Since the 1990s, the neoliberal movement has accelerated the privatisation of social services, framing disability inclusion as a cost rather than a shared obligation. This economic marginalisation is combined with geographical exclusion, as inaccessible workplaces and limited employment opportunities prolong cycles of poverty and reliance.

Grassroots initiatives and community-based organisations are actively using decolonial strategies to redefine belonging. Initiatives to educate *n’angas* [traditional healers] as advocates for children with disabilities demonstrate unique approaches to combining indigenous knowledge systems with modern rights-based frameworks (Chataika 2012). The rise of Ubuntu-inspired peer mentorship activities has exciting prospects for fostering social inclusion and addressing stigma (Marongwe 2019). These projects demonstrate efforts to prioritise indigenous epistemologies while overcoming systemic barriers to inclusion.

Language and image play critical roles in fostering a sense of belonging. The ongoing use of derogatory terms for disability in Shona and Ndebele languages reflects established cultural beliefs that promote marginalisation. Initiatives targeted at improving positive portrayals of disability in media and literature, on the other hand, offer potential for influencing public perceptions and fostering a more inclusive national identity.

Antonsich’s (2010) theoretical framework helps to understand the underlining factors that impact PWD belongingness in Zimbabwe. The theory of belonging is both a profound personal connection to community and a political struggle against colonial legacies and current forms of exclusion. Decolonial approaches for disability justice must have two components: encouraging Ubuntu-inspired belonging and dismantling systemic inequities to reconceptualise citizenship as truly inclusive.

Anticipating the future, the path to transformative inclusion requires a diverse strategy. Policy improvements must go beyond cosmetic representation to ensure that PWD are fully involved in decision-making processes at all levels of government. Educational curricula must be updated to integrate diverse representations of disability, leveraging indigenous knowledge systems and modern rights-based frameworks. Infrastructure construction must prioritise universal design principles to create physically accessible places that foster a sense of communal belonging.

## Research methods and design

This study, through a qualitative desktop technique, meticulously examines current textual material to investigate intricate socio-cultural occurrences (Bowen 2009; Flick 2014). This approach is based on interpretive traditions like constructivism. It sees social and cultural events as constructed through meaning-making. Because of this, it requires a careful look at documents in their specific historical and multifaceted contexts, production, content and reception to find the subtle layers that shape the reality being represented. Peer-reviewed articles, policy documents, non-governmental organisation (NGO) reports, media archives and grey literature, including dissertations and conference proceedings published between 2000 and 2023, were collected for the study.

Selective sampling focuses on resources that are most relevant to Zimbabwe’s particular disability environment, such as its policies, cultural norms and service realities. This makes sure that the study topic is as relevant as possible (Liamputtong 2013; Ravitch & Carl 2021). This focuses on the most important data. While NGO publications (e.g. UNICEF, Leonard Cheshire Disability Zimbabwe [LCZ]) offered grassroots viewpoints, documents including the 2013 Constitution and the 2021 National Disability Policy came from official sources. Media pieces (such as ZimLive) provided real-time narratives of discriminatory policies. Zimbabwean literature offers complex portrayals of disability and society, affirming the personhood of PWD (Vera 2008). Inclusion criteria required that documents be published in English, Shona, or Ndebele (with translations provided where necessary) and focus on disability, spirituality, or citizenship in Zimbabwe or the Southern African region. Except for key colonial-era literature like the *1959 Mental Disorders Act*, exclusion criteria removed research missing intersectional analysis (e.g. gender-neutral frameworks) and pre-2000 sources. Data analysis was done thematically.

As a desktop study, ethical concerns focused on the polite portrayal of disadvantaged views. Where sources allowed, direct quotes from sensitive groups, including LGBTQ+ people with disabilities, were anonymised. Analysing painful stories like accounts of sexual abuse by contextualising systematic sources of harm instead of sensationalising them would help to show sensitivity (Southern Africa HIV and AIDS Information Dissemination Service [SAFAIDS] 2022). Grassroots-authored studies like those of Deaf Women Included (DWI 2003) were given top priority to overcome this restriction, and results were triangulated across several sources, for example, comparing policy rhetoric with media-reported realities.

## Results and discussion

### Cultural, indigenous cosmologies and disability

Disability [*urema*] is deeply intertwined with spiritual and moral issues in Shona and Ndebele cosmologies, reflecting complex ideas about belonging and exclusion (Dube 2020). These indigenous frameworks offer both opportunities and challenges to PWD negotiating their position in Zimbabwean culture.

#### Ancestors, mhondoro and moral causality

According to Shona customs, bodily ailments such as lameness [*kuremara*] are caused by ancestor discontent [*vadzimu*], and failure to fulfil ritual tasks is considered a curse. This is not a general position, though; women from poor backgrounds or those with disabilities often experience compounded exclusion, highlighting how disability interacts with gender and class (SAFAIDS 2022). Although this term may stigmatise, it also provides a unique opportunity for communal integration and agency.

In Shona cosmology, disability is understood by spiritual tales oscillating between respect and shame. For example, Mhondoro, spirit mediums with congenital disabilities, are regarded as ancestral intermediaries, resolving clan conflicts in Buhera District (Chataika 2012:389). This spiritual function helps people with disabilities to feel place-belonging and to be included in society.

By seeing disability as a shared duty, Ubuntu ethics (unhu) offset this ambivalence. For instance, zvigure networks in Mwenezi District use community resources to assist families with a member who has a disability, thereby operationalising Ubuntu’s interdependence (Marongwe 2019:45). But neoliberal policies commercialise care, weakening these spiritual–communal ties (Grech 2015:48). Such opinions reflect the continued stigma and ignorance around disability. Disability support is framed by neoliberal policies as a financial burden, thus undermining Ubuntu’s ethic of shared responsibility (Grech 2015).

### Colonial disruptions of indigenous systems

#### Missionaries, schools for persons with disabilities and epistemic violence

Indigenous disability systems saw great upheaval during the colonial era. Under the colonial rationale of ‘civilising’ children with disabilities, missionaries such as John White of Dadaya Mission (founded 1919) segregated them into residential schools. According to colonial records from 1932, the Native Affairs Department denigrated PWD Africans to mission colonies, denied them land rights and labelled them as unfit for tax.

Policies following independence have battled to completely decolonise responses to disability. Aiming for better conditions for PWD in Zimbabwe, the *Disability Act* kept elements of colonial logic by giving institutionalisation priority over community integration (*Disability Act, 1992*).

However, in modern Zimbabwe, there is a continuous clash between Western biomedical practices and indigenous knowledge systems. According to a 2021 Mashonaland Central survey, 70% of families consulted n’angas before seeking biomedical therapy for disability; nonetheless, the Ministry of Health excludes traditional healers from policy talks (Marongwe 2019). This epistemic marginalisation has an impact on education; as recently as 2020, the Ministry of Primary Education was chastised for removing Shona folktales featuring PWD as heroes from textbooks, effectively eliminating indigenous perspectives on disability from mainstream curricula (Ndlovu 2022).

Despite these challenges, grassroots groups are working to connect modern rights-based approaches to disability inclusion with indigenous knowledge systems. In Chiweshe, a programme that teaches *n’angas* to advocate for impoverished children’s education increased school enrolment by 30% (Chataika 2012). Such projects demonstrate how ancient rituals can be creatively adapted to enhance PWD’s political activism and sense of place.

The African Network for Evidence-to-Action, founded in 2007, represents a purposeful attempt to restore the dignity and identity of individuals with disabilities in Africa through the Ubuntu idea (Miji, MacLachlan & Mji 2011). This network aims to build a diverse community of practice that provides evidence and ideas for improving the lives of PWD. It emphasises interdependence and reciprocal accountability.

Scholars like Chisale (2020) argue that inclusion of PWD is one of the core values of Ubuntu. This viewpoint calls into question the dichotomies that can lead to fear, stigma, and prejudice against persons who depart from society’s expectations. Rather, it increases our understanding of humanity as part of a web of life in which ability-based discrimination is not tolerated.

The road forward necessitates a multifaceted strategy that values indigenous knowledge while also addressing contemporary issues. Policy adjustments must go beyond tokenistic representation to ensure that disabled persons are meaningfully involved in decision-making processes at all levels of government (Eide & Ingstad 2013). Using both indigenous knowledge systems and current rights-based methodologies, educational curricula should be modified to integrate multiple representations of disability.

Dealing with the financial consequences of exclusion is vitally necessary. Targeted job programmes, easily accessible microfinance ventures and social protection programmes tailored to the needs of PWD can help break the cycle of poverty and reliance (Mitra, Posarac & Vick 2013). Efforts to eliminate stigma and discrimination must include religious and traditional leaders as primary advocates for inclusive community standards (Swartz et al. 2018).

As Zimbabwe grapples with its colonial legacy and present socioeconomic issues, PWD’s experiences give a unique lens through which to evaluate broader questions of belonging, citizenship and social justice. Zimbabwe could build a more inclusive society that recognises the numerous contributions made by all of its people by embracing Ubuntu ideals and critically interacting with both indigenous and modern approaches to disability (Chataika et al. 2015).

### Spirituality and religion: Contested spaces for disability belonging

#### Rituals, stigma and N’angas as mediators

In Zimbabwe, the intersection of spirituality, religion and disability creates a complex landscape in which the sense of belonging is constantly reinterpreted. African traditional religions (ATRs) and various Christian denominations manage disability through behaviours that alternate between inclusion and exclusion, creating problematic contexts for people with disabilities.

In Zimbabwe, ATRs commonly interpret disability from a spiritual perspective, with rituals having a large impact on communal reactions. In Mberengwa, *n’angas* [traditional healers] perform *kurova guva* [ancestral appeasing] rites for albino children, which combine herbal medicines with invocations to *vadzimu* [ancestral spirits] (Dube 2020). These exercises represent a complete approach to disability that considers both spiritual and physical components. Nonetheless, these rituals can sometimes reinforce stigma and foster negative attitudes towards disability.

Some Gutu households first hid children with cerebral palsy because of concern for witchcraft allegations (LCZ 2019); however, Ingstad (1995:376) issues a warning against generalising such situations. As evidenced by Chiweshe’s community-led education projects, many Zimbabwean families instead use Ubuntu ideals to fight for the inclusion of children with disabilities (Chataika 2012:391). This demonstrates how spiritual interpretations of sickness can lead to social isolation and marginalisation. In contrast, other communities are reinterpreting ATRs to encourage diversity. In Chiweshe, a 2022 programme educated n’angas to encourage the education of children with disabilities, resulting in a 30% increase in local school enrolment (Chataika 2012). This paradigm combines indigenous knowledge systems with current rights-based frameworks to promote disability inclusion.

Traditional leadership has an important role in shaping communal perceptions of disability. In 2021, Chief Nyangazonke of Zvimba District declared disability to be a communal responsibility, requiring village leaders to set aside land for families with PWD (Marongwe 2019). Still, inclusion is not equal: LGBTQ+ people with disabilities negotiate exacerbated stigma, while wealthy metropolitan attendees could find support systems not accessible to rural colleagues (Gays and Lesbians of Zimbabwe [GALZ] 2021). Such declarations can have a significant impact on local attitudes and practices. Although Ubuntu’s ethics see disability as a shared burden, colonial and neoliberal influences have warped indigenous systems, therefore allowing exclusive interpretations. Spiritual stories formerly respected *mhondoro* [spirit mediums]; explanations that stigmatise disability as ‘ancestral punishment’ (Chataika 2012:389; Dube 2020:54). These paradoxes draw attention to the flexibility of indigenous knowledge: depending on sociopolitical settings, it could empower or marginalise.

#### Sin, exorcisms and prosperity gospel pressures

As Zimbabwe confronts economic difficulties and societal change, the experiences of PWD in spiritual and religious contexts provide significant insights into broader themes of belonging, citizenship and social justice. The conflict among traditional beliefs, Christian doctrines and modern rights-based perspectives on disability underscores the necessity for continuous discourse and education.

The Christian majority in Zimbabwe, particularly within Apostolic sects, typically sees disability as a problem of sin and redemption. The Mapostori sect, which makes up a significant portion of society, attributes disabilities to generational curses, prompting fasting and exorcisms (Chikwaiwa 2020). This mindset may lead to negative acts and increased marginalisation of people with disabilities. In 2019, a Harare Apostolic church gained headlines for detaining a small child with epilepsy during all-night prayers, claiming the objective was to expel demons (ZimLive 2019). Such incidents underscore the critical need for education and awareness within religious communities.

Mainstream churches usually combine donation and advocacy in their response to disability. The Catholic Church in Gweru established St. Catherine’s School for the Deaf in 1962, making it Zimbabwe’s first institution to offer sign-language education. Nonetheless, the institution faces significant challenges because of the country’s economic crisis. From 2015 to 2020, enrolment declined from 200 to 80 pupils because of hyperinflation (Musengi 2021). This reduction highlights the vulnerability of disability-inclusive organisations to broader socioeconomic forces.

Pentecostal denominations, such as the Zimbabwe Assemblies of God Africa, promote faith healing as a treatment for illness. This technique may bring hope for some, but it also risks exacerbating marginalisation. The prosperity gospel, which is spread in various churches, can put undue pressure on attendees with disabilities. According to a 2021 poll, 62% of congregants with disabilities in Bulawayo were pressured to donate miracles, which exacerbated their financial issues (Amin, Kpobi & Swartz 2021). This case exemplifies the interaction of religious beliefs and economic hardship, worsening the marginalisation of PWD.

Notably, syncretic practices (blending of different religious beliefs) are emerging that aim to reconcile ATR and Christian views on disability. In Masvingo, certain Apostolic organisations use *kubatana* [unity] rites, in which members with disabilities pray as living testimonies of God’s grace (Chikwaiwa 2020). These practices suggest the prospect of more inclusive religious environments that value the contributions of people with disabilities.

The complex link between disability, spirituality, and religion in Zimbabwe reflects broader cultural viewpoints and issues. Although certain habits and attitudes contribute to the marginalisation of PWD, there are growing initiatives aimed at promoting inclusion and overcoming stigma. Religious and traditional authority have a huge influence in shaping communal perceptions, as evidenced by Chief Nyangazonke’s proclamation in Zvimba District.

It is imperative to endorse projects that foster inclusive religious and spiritual activities while honouring cultural traditions. This may entail more training for religious leaders regarding disability rights, integrating disability awareness into religious education curricula and enhancing the representation of individuals with disabilities within religious communities. By examining the intricate intersections of spirituality, religion, and disability, Zimbabwe may cultivate more inclusive environments where all individuals, irrespective of ability, can experience belonging and acceptance.

### Belonging and exclusion in families and communities

#### Hyperinflation, malnutrition and school dropouts

Families and communities are very important in Zimbabwe for the care and inclusion of PWD, although this dynamic is complex with conflicts between belonging and exclusion. While Ubuntu ethics and grassroots projects provide means for resilience and inclusion, economic difficulties, cultural stigma and poor infrastructure aggravate the marginalisation of PWD.

Families continue to be the major carers for PWD, but the financial crisis has made it more difficult for them to give enough help. Forty-five per cent of children with disabilities in rural regions are malnourished because of carers’ inability to buy specialised diets, according to a 2022 Zimbabwe National Statistics Agency (ZimStats) survey (ZimStats 2022). Hyperinflation peaked at 700% in 2020, forcing families to make severe survival trade-offs. Family dynamics are not homogeneous: although rural families with low means must make more difficult trade-offs, educated urban families may use NGOs for help (Matsika 2017). Parents in Chiredzi said they pulled children with disabilities from school to save $50.00 a month on travel expenses (Matsika 2017). Many times, these financial restrictions cause abled siblings to be prioritised, thereby further marginalising family members with disabilities.

Indigenous cultural practices make mothers of children with Down syndrome excluded from communities. In Mutare, mothers with children with Down syndrome are sometimes branded as *varoyi* [witches]; their lineage is cursed. This social isolation separates carers and supports unfavourable views of disability. Likewise, among the Shona people, cultural ideas may dehumanise PWD. Derived from the core word *rema* [weak or frail], the term *chirema* captures society’s perceptions that view PWD as unable to significantly influence family or community life (Chengeta & Msipa 2012). These stereotypes support exclusion and restrict chances for PWD to engage in social events, jobs, or education.

#### Zvigure systems and community-based rehabilitation

Notwithstanding these obstacles, Ubuntu principles offer a contrast by encouraging group accountability and shared compassion. The *zvigure* [extended family] system in Mwenezi District epitomises this value. A 2019 case study focused on a grandmother tending to her grandson with disabilities and how neighbours and the community donated maize (Marongwe 2019). Such behaviours show how Ubuntu preserves place-belongingness even in difficult economic times and confirm emotional ties to shared environments.

Communities also apply Ubuntu ideas to grassroots projects. Non-governmental organisations like Zimcare Trust teach households income-generating activities like soap-making for mothers of children with disabilities. While encouraging social participation, these initiatives improve economic resilience (LCZ 2019). Community-driven support is consistent with documented models of community-based rehabilitation in rural Zimbabwe (Choruma 2007). A community-based rehabilitation initiative teaches residents of rural Chimanimani to build ramps out of locally available materials, thus enhancing access to clinics for wheelchair users (Chataika 2012). These attempts, meanwhile, are sensitive to outside shocks. Six hundred PWD from Chipinge were displaced by Cyclone Idai in 2019; many of them now live in temporary shelters without enough support systems or assistive tools (Zimbabwe Parents of Children with Disabilities Association [ZPCDA] 2021).

Harare and other metropolitan regions show striking cases of socio-spatial segregation. Only 12% of public buses have ramps; vendors or poorly maintained infrastructure block footpaths. Wheelchair users must rely on unofficial assistants, a service costing $2.00 per use in an environment of extreme unemployment, after a 2023 audit of Mbare Market identified no accessible restrooms (Maunganidze 2020; Ndlovu 2022). These barriers physically separate PWD from public areas and undermine their sense of inclusion.

Crises like droughts or diseases worsen the fragility of belonging. In such situations, spiritual frameworks can weaponise disability. For instance, PWD were shunned as harbingers of death, therefore distorting their sense of home and community during the 2008 cholera outbreak in Chitungwiza (Dube 2020). These events show how cultural beliefs and socioeconomic demands interact to intensify exclusion.

Also, colonial legacies disrupted the indigenous systems of family caring and belonging. British colonial officials destroyed conventional support systems and enforced Eurocentric ideas that denigrated disabilities. For instance, Shona folktales with PWD heroes were deleted from the curriculum in line with more general attempts to eradicate indigenous knowledge systems (Ndlovu 2022). Denying cultural status to PWD, this epistemic aggression strengthened exclusive narratives.

Still, Ubuntu philosophy presents a transforming paradigm for the reinterpretation of disability inclusion. Rooted in ideals of interconnectedness and communalism, Ubuntu underlines that every member of society, regardless of ability, is linked and deserving of dignity and care (Chisale 2020). Community-driven projects combining ancient methods with contemporary disability campaigning clearly show practical uses of Ubuntu. Some rural towns, for example, have reinterpreted ceremonies like *kurova guva* [ancestral appeasement] to incorporate lobbying for access to schooling for children with disabilities (Chataika & McKenzie 2013).

Although these illustrations show the possibility for inclusive policies based on Ubuntu ethics, systematic obstacles are still somewhat common. Dealing with these issues calls for a multifarious strategy combining family financial support with more general changes in society’s perceptions of disability. Policies must provide accessibility in urban infrastructure as a top priority, then support grassroots initiatives using conventional knowledge systems for inclusive development.

Families and societies in Zimbabwe thus negotiate difficult dynamics of belonging and exclusion for PWD. Although cultural shame and economic breakdown worsen marginalisation, Ubuntu ethics provide means for resilience by encouraging group care and shared accountability. Zimbabwe may help to build an inclusive society where everyone has the chance to flourish by including these ideas in policy agendas and community projects.

### Citizenship, intersectionality and disability rights in Zimbabwe policy frameworks

#### From the *1959 Mental Diseases Act* to the 2021 National Disability Policy

Although the 2013 Constitution guarantees disability rights, its application resembles what El-Lahib (2015:615) defines as ‘charity-model paternalism’, hence replicating colonial logics of reliance (Meekosha 2011:7). By centring intersectionality (Crenshaw 1989), grassroots groups such as DWI oppose this in their 2023 #DeafRightsMatter campaign (DWI 2023).

The National Disability Board, established by the 2013 legislation, illustrates the chronic underfunding that afflicts disability rights programmes. Policies must address multilayer marginalisation: rural women need customised healthcare access, and LGBTQ+ people with disabilities need legislative safeguards outside of general disability rights (Muchegwa 2023; SAFAIDS 2022). This underscores the need for intersectional approaches to disability policy, as argued in regional advocacy frameworks (Mpofu & Harley 2018). In 2022, the Board, constrained by a $50 000 budget, lacks the capacity to perform extensive statewide audits or adequately oversee policy implementation (Ndlovu 2022). This budgetary limitation significantly restricts the Board’s capacity to execute its purpose and champion the rights of individuals with disabilities.

Accessibility of public infrastructure continues to be a substantial obstacle to inclusion. A 2023 report from the Harare City Council indicated that merely three of 150 government offices are equipped with lifts, underscoring the widespread physical exclusion encountered by those with disabilities in accessing public services (ZEC 2023). The deficiency in accessibility permeates educational institutions, where, notwithstanding inclusive education policies, 78% of educators lack expertise in special needs education. This gap highlights persistent challenges in implementing inclusive education, a concern extensively documented in the Zimbabwean context (Phasha & Myaka 2014). The circumstances at Zengeza High School in Chitungwiza, where deaf students are compelled to share textbooks with 50 classmates, exemplify the critical resource limitations and insufficient assistance for students with disabilities (Musengi 2021).

The right to political participation, a fundamental aspect of citizenship, continues to be unattainable for some PWD in Zimbabwe. This structural obstacle to voting rights highlights the pressing necessity for extensive electoral changes to guarantee equitable access and participation for all citizens.

#### Deaf Women Included, Zimbabwe Parents of Children with Disabilities Association and intersectional gaps

Advocacy organisations such as the National Council of Persons with Disabilities of Zimbabwe (NCDPZ) have achieved notable advancements in promoting enhanced inclusion. The changing role of non-governmental organisations in disability advocacy in Southern Africa has been analysed in relation to shifting development paradigms (Peters 2019). Their successful 2022 High Court verdict requiring sign-language interpreters at press conferences signifies progress in accessibility (NCDPZ 2022). Political apathy among voters with disabilities persists, with merely 12% engaging in the 2023 elections, attributing their reluctance to concerns about harassment at polling sites (ZimStats 2022). This low attendance signifies both the physical obstacles to voting and the pervasive cultural prejudices that persist in marginalising PWD.

The heritage of institutions such as Jairos Jiri, established in 1950, exemplifies the intricate history of disability services in Zimbabwe. Early institutions such as Nguboyenja in Bulawayo, while offering vocational training and support, often reinforced isolation and reflected colonial paternalistic views towards those with disabilities (Mpofu 2003). The 2020 testimonials presented to the Zimbabwe Human Rights Commission (ZHRC), which elucidate the coerced labour at Jiri’s workshops throughout the 1970s, underscore the historical maltreatment endured by PWD in Zimbabwe (ZHRC 2020). Current improvements within these institutions exhibit inconsistent advancement; the inclusion of children with disabilities in mainstream classrooms at Kadoma School starkly contrasts with the overcrowded conditions at the Gwanda dormitory, where 40 people share two bathrooms (LCZ 2021).

Intersectional methodologies regarding disability rights are increasingly being embraced by organisations such as DWI, established in 2017. The sign-language literacy initiative in Mufakose, which has taught 200 women, exemplifies the efficacy of focused interventions in enhancing access to vital services such as maternal healthcare (Muzenda 2021). Deaf Women Included’s partnership with Parliament in formulating the *Sign Language Act*, notwithstanding delays caused by budgetary reductions, signifies a vital advancement towards the legal acknowledgement of sign language (NCDPZ 2022). The #DeafRightsMatter campaign has highlighted workplace discrimination, with a 2023 poll indicating that 89% of deaf women are unemployed, frequently because of employer biases, including the perceived inability to answer phones (DWI 2023).

The compounded marginalisation experienced by women with disabilities is especially concerning. Individuals identifying as LGBTQ+ and possessing disabilities encounter heightened risks, as evidenced by a 2021 survey conducted by GALZ, which revealed that 60% of LGBTQ+ members with disabilities had been subjected to blackmail, frequently because of the apprehension of their sexual orientation being disclosed (Gunda 2020).

Notwithstanding these limitations, grassroots initiatives are redefining inclusion via creative methodologies. The 2020 campaign by the ZPCDA in Mutoko, which educated 50 traditional healers [*n’angas*] to dispel beliefs surrounding disabilities, led to a 40% decrease in school dropout rates among children with disabilities (Chataika 2012). This method illustrates the capacity for merging cultural traditions with disability rights campaigning. The Ubuntu Disability Network’s 2023 peer mentorship programme in Hwange pairs children with disabilities with elders to rejuvenate indigenous knowledge systems and foster intergenerational support (Marongwe 2019). These grassroots initiatives illustrate the promise of decolonial strategies for disability inclusion that prioritise indigenous knowledge systems and community-led solutions. By integrating traditional practices with modern rights-based frameworks, such programmes provide a pathway to more culturally relevant and sustainable forms of inclusion.

Zimbabwe’s struggle with the execution of its disability legislation underscores the pressing necessity for a comprehensive strategy for inclusion, as evidenced by the experiences of PWD. This strategy must consider legal and policy frameworks, societal attitudes, physical infrastructure and the intersectionality of discrimination. By elevating the voices of PWD and endorsing community-driven projects, Zimbabwe can foster a more inclusive society that appreciates the varied contributions of all its inhabitants.

## Conclusion

This study shows that disability in postcolonial Zimbabwe is a place of disputed belonging, a conflict between spiritual respect and exclusion, shaped by colonial legacies and neoliberal austerity. Although indigenous cosmologies interpret disability as ancestral communication, for example, *mhondoro* spirit mediums, this respect remains conditional, dependent on preserving communal peace. Such uncertainty captures more general postcolonial paradoxes. While neoliberal policies privatise welfare, hence reducing disability inclusion to a financial burden, Ubuntu ethics support collective care.

Although it highlights institutional exclusion, Antonsich’s theoretical framework falls short in capturing the way spiritual and financial marginalisation interact in African settings. For example, LGBTQ+ people with disabilities experience exclusion brought on by both heteronormative governmental regulations and social stigma. This emphasises how limited Eurocentric belonging theories are in postcolonial environments and how decolonial methods centred on indigenous epistemologies are thus needed.

The most important contribution of the study comes in revealing the paradox of progressive legislation. While inaccessible voting booths disenfranchise voters with visual impairments, Zimbabwe’s 2013 Constitution protects disability rights, therefore upholding colonial-era politics of belonging. Transformative inclusion thus calls for the destruction of these legacies by means of participatory governance, that is, including *dandaro* [community dialogues] into policymaking and allocating funds to grassroots movements like DWI, thus bridging rights-based activism and Ubuntu ethics. Future studies should explore how global neoliberal goals compromise localised disability justice, especially for rural women and LGBTQ+ groups. Scholars may go beyond colonial binaries and rethink belonging as an inherent right, not a conditional privilege, by centring people with disabilities as knowledge producers.
